# A healthy individual with a homozygous *PTCH2* frameshift variant: Are variants of *PTCH2* associated with nevoid basal cell carcinoma syndrome?

**DOI:** 10.1038/s41439-019-0041-2

**Published:** 2019-02-22

**Authors:** M. Altaraihi, K. Wadt, J. Ek, A. M. Gerdes, E. Ostergaard

**Affiliations:** Department of Clinical Genetics, Copenhagen University Hospital Rigshospitalet, Copenhagen, Denmark

**Keywords:** Cancer genomics, Risk factors

## Abstract

Variants in *PTCH2* have been described to be associated with Nevoid Basal Cell Carcinoma Syndrome (NBCCS). We report a family with a healthy female who is homozygous for a frameshift variant, c.269delG, p.(Gly90Alafs*4), in *PTCH2* and her heterozygous daughter. The variant predicts a frameshift and a premature stop codon. A summary of reported heterozygous individuals with germline *PTCH2* variants along with the existence of a healthy homozygous individual question whether variants in *PTCH2* are associated with NBCCS.

The Patched 2 (*PTCH2)* gene and its close homolog, *PTCH1*, both encode transmembrane receptors of the patched gene family in the sonic hedgehog pathway^1^. Variants in the *PTCH1* gene have been found to cause nevoid basal cell carcinoma syndrome (NBCCS), also known as Gorlin syndrome, with typical clinical manifestations of multiple basal cell carcinomas, odontogenic keratocysts of the jaw, palmar and plantar pits, intracranial ectopic calcification, facial dysmorphism (macrocephaly, cleft lip/palate), and eye anomalies (cataract, pigmentary changes of the retinal epithelium and developmental defects)^2,3^. According to the NBCCS criteria of Evans et al., at least two major and one minor criteria or one major and three minor criteria must be fulfilled for a patient to be diagnosed with NBCCS^2,3^. Autosomal dominantly inherited NBCCS is transmitted with complete penetrance and variable expressivity^4,5^. Large epidemiological studies of NBCCS report different ethnicity-dependent penetrances of NBCCS: the most significant is the lower prevalence of basal cell carcinoma in Japanese NBCCS patients over 20 years of age (51.4%) compared to American NBCCS patients (91%), Australian NBCCS patients (85%), and British NBCCS patients (73%)^2,6–8^. It has been suggested that *PTCH2* variants can also cause NBCCS, albeit with a milder phenotype^9^.

PTCH1 plays an important role in the sonic hedgehog pathway, which is a regulator of patterning and development in the embryo and adult. In brief, the pathway comprises the sonic hedgehog ligand (Shh), PTCH1/PTCH2 transmembrane receptors and the G protein-coupled receptor Smoothened (Smo). The best-studied member of the patched gene family, the PTCH1 receptor, suppresses the release of Smo, but when Shh binds to the PTCH1 receptor, this inhibition is alleviated, resulting in Smo activation and translocation from the plasma membrane to the primary cilium. While PTCH1 is the primary Shh receptor, PTCH2 has a minor compensatory role in Shh signal transduction, although its function is yet to be fully understood^10^. Deficient PTCH1 and PTCH2 receptors decrease the inhibition of Smo, which leads to increased activity of transcription factors^11^.

We here present a healthy female homozygous for a *PTCH2* frameshift variant, and we therefore question the association between *PTCH2* variants and NBCCS.

The clinical investigations were conducted according to the principles expressed in the Declaration of Helsinki. Both patients gave oral and written informed consent for publication. Since the investigations were performed as part of a diagnostic workup, we did not apply for ethics approval.

Patient 1: An 18-year-old woman of Middle-Eastern descent with consanguineous parents (1st cousins once removed) was diagnosed with severe hypermobile Ehlers-Danlos syndrome (EDS), congenital scoliosis, epilepsy, and asthma at the age of 2 years (Fig. 1a). During the following years, as a likely consequence of EDS, she underwent three umbilical hernia operations. At 14 years old, she was diagnosed with sleep apnea and began using autoCPAP as treatment during the nights. Additionally, she suffered from joint instability, which resulted in nearly chronic subluxations of the bilateral carpometacarpal joints. She was using orthotics and a walker to maintain balance and stability while walking. Furthermore, the patient was treated for chronic rhinosinusitis, weakened mastication, and difficulty in swallowing. Lastly, the patient had also been affected by migraine and fluctuations of eye accommodation and refraction. To our knowledge, she did not have any of the symptoms included in the diagnostic criteria for NBCCS. Whole-exome sequencing was performed on blood from the patient.

**Fig. 1:**
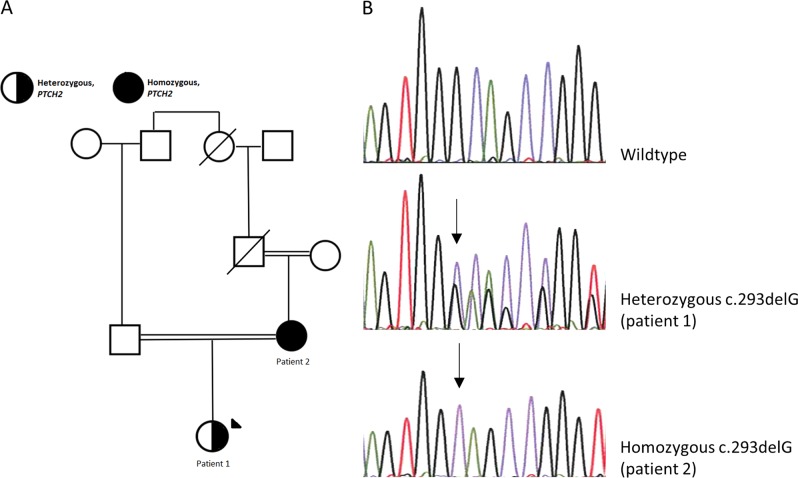
Pedigree and electropherogram of the family with a *PTCH2* variant. **a** Pedigree of the family with a *PTCH2* variant. **b** Electropherogram of the *PTCH2* c.269delG variant showing wild-type, heterozygous (patient 1) and homozygous (patient 2) individuals

Patient 2: The 39-year-old mother of patient 1 had been diagnosed with hypermobility syndrome at the age of 34 years. Furthermore, she had been affected with recurrent respiratory infections with hemoptysis for several years in her early thirties. She reported no NBCSS manifestations and was further examined for three major NBCCS criteria. Skin examination by a dermatologist revealed no basal cell carcinomas or palmar or plantar pits, and a dental CT scan showed no cysts of the jaw. We refrained from exposing the patient to unnecessary radiation, and hence she was not examined for the fourth major criterion, calcification of the falx cerebri. She did not have first-degree relatives affected with NBCCS, which is the fifth major criterion. To our knowledge, she did not fulfill any of the minor criteria for NBCCS.

Variant analysis of whole-exome sequencing data showed no pathogenic variants explaining the severe Ehlers Danlos syndrome in patient 1. However, as an incidental finding, a heterozygous variant, c.269delG, p.(Gly90Alafs*4), was identified in exon 3 of *PTCH2*, leading to a frameshift and a premature stop codon. Sanger sequencing of DNA from her mother showed homozygosity for the c.269delG, p.(Gly90Alafs*4) variant (Fig. 1b).

To our knowledge, the *PTCH2* variant has not been described previously, and it is not reported in ClinVar (https://www.ncbi.nlm.nih.gov/clinvar/) or the Genome Aggregation Database (gnomAD)(http://gnomad.broadinstitute.org), which comprises more than 245,000 alleles in this region.

Patient 2 with the homozygous *PTCH2* variant had worn traditional ethnic clothing covering the arms, legs, and hair from a young age, and she had darkly pigmented skin. Only her face and hands were exposed to ultraviolet radiation, and hence the risk of developing basal cell carcinomas could be considered low. However, ~80% of reported basal cell carcinomas are located in the face, head, and neck, and her risk of basal cell carcinoma is therefore not negligible^12^.

It is notable that patient 1 is affected with scoliosis, as 40% of NBCCS cases are reported to have scoliosis^13^, but this is not significant as scoliosis can occur as part of many syndromic diseases.

Previously, three papers have described eight individuals, including six belonging to one family, with heterozygous *PTCH2* variants (Table 1). Six individuals had palmar or plantar pits, three individuals had keratocysts of the jaw, two had bifid ribs, and one individual had multiple basal cell carcinomas. A newborn had embryonal rhabdomyosarcoma^14,15^. None of the 10 *PTCH2* individuals listed in Table 1 fulfilled the diagnostic criteria for NBCCS proposed by Evans et al.^3^, whereas using the diagnostic criteria proposed by Kimonis et al., 7 of the 10 individuals with *PTCH2* variants would be diagnosed with NBCCS, as these criteria demand only that two major criteria be fulfilled^7^.

**Table 1:** Summary of *PTCH2* literature reports

Reference	Present report (patient 2)	Present report (patient 1)	Taeubner (16)	Fujii^9^	Fan^14^	Fan	Fan	Fan	Fan	Fan
PTCH2 variant	c.269delG (homozygous)	c.269delG (heterozygous)	c.1864C>T (rs11573586) (heterozygous)^a^	c.1172_1173delCT (rs777588680) (heterozygous)	c.2157G>A (rs121434397) (heterozygous)	c.2157G>A (heterozygous)	c.2157G>A (heterozygous)	c.2157G>A (heterozygous)	c.2157G>A (heterozygous)	c.2157G>A (heterozygous)
Variant type	Frameshift	Frameshift	Missense (p.(His622Tyr))	Frameshift (p.(Ser(391Ter))	Missense (p.(Arg719Gln))	Missense	Missense	Missense	Missense	Missense
*In silico prediction of pathogenicity*										
SIFT			Deleterious (0.01)		Tolerated (0.19)					
MutationTaster			Probably damaging (0.978)		Probably damaging (0.999)					
Polyphen2			Benign (0.10)		Benign (0.33)					
CADD score			1.9		3.6					
Allele frequency in gnomAD	–	–	2683/276,572 alleles (26 homozygous)	65/277,190 alleles (1 homozygous)	8/276,184 alleles (0 homozygous)					
Sex	F	F	M	F	M	F	F	F	M	F
Age (years)	39	18	0	13	45	57	42	40	17	25
Ethnicity	Middle Eastern	Middle Eastern	Turkish	Japanese	Chinese	Chinese	Chinese	Chinese	Chinese	Chinese
*Major NBCCS criteria*										
Basal cell carcinoma	No	–	No	No	No	Yes (multiple)	No	No	No	No
Keratocysts of the jaw	No	–	No	Yes	No	No	No	No	No	Yes
Palmar or plantar pits	No	–	No	No	Yes	Yes	Yes	Yes	Yes	Yes
Ectopic calcification	–	–	No	No	No	No	No	No	No	No
First-degree relative with NBCCS	No	No	No	No	No	No	No	No	No	No
Number of major criteria fulfilled	0	0	–	1	1	2	1	1	1	2
Number of minor criteria fulfilled	0	0	–	1 (bifid ribs)	0	0	0	0	1 (bifid ribs)	0
Other	No	No	Embryonal rhabdomyosarcoma	No	No	No	No	No	Yes	No
NBCCS (Evans criteria)	No	No	–	No	No	No	No	No	No	No
NBCCS (Kimonis criteria)^b^	No	No	–	Yes	Yes	Yes	Yes	Yes	Yes	Yes

^a^This individual also carries a *PTCH1* variant.

^b^As opposed to the criteria of Evans et al., Kimonis et al. define bifid ribs as a major criterion and require only two major criteria or one major and two minor criteria for a patient to be diagnosed with NBCCS.

The reported *PTCH2* variants (Table 1) included one 2-bp deletion resulting in a frameshift and two missense variants. One of the missense variants, c.1864C>T, p.(His622Tyr), was found together with a *PTCH1* variant in a newborn with rhabdomyosarcoma, for whom no follow-up was reported. This *PTCH2* missense variant is very frequent in gnomAD, with 26 homozygous individuals reported, and in silico programs give a low probability of the variant being pathogenic. The other missense variant (c.2156G>A, p.(Arg719Gln)) was reported in six members of one family, of whom none had NBCCS according to the Evans criteria but all fulfilled the Kimonis diagnostic criteria. The variant was reported in eight individuals in gnomAD, and in silico predictions do not support its pathogenicity. The frameshift variant, c.1172_1173delCT, was found in 65 alleles, including one homozygous individual.

A variety of *PTCH1* variants cause NBCCS, including missense, nonsense, frameshift, and splice site variants. The gnomAD database reports 27 different loss of function (LOF) variants in *PTCH1* (transcript size 8057 bp) and 85 LOF variants in *PTCH2* (transcript size 4298 bp), of which one was reported as homozygous (/mentary table S1). Assuming the variants are not on the same alleles, these LOF variants are found in 59 individuals in *PTCH1* and in 480 individuals in *PTCH2*; hence, LOF variants seem to be well-tolerated in *PTCH2*.

Analyses of knockout (KO) mice revealed that *PTCH1* ^−^/^−^ mutants were lethal at embryonic day 9.5 due to hyperactivation of Shh signaling. By contrast, *PTCH2* ^−^/^−^ mutants were viable, fertile and apparently normal; however, alopecia, epidermal hyperplasia, dermal hyperplasia, hair follicle loss, and ulceration were frequently observed with progressing age in *PTCH2* ^−^/^−^ mutant male mice, whereas *PTCH2* ^−^/^−^ mutant female mice did not show any abnormalities. Neither male nor female *PTCH2* double KO mice developed basal cell carcinomas^10,16^

Homozygosity for a frameshift variant in the *PTCH2* gene does not seem to have caused any NBCCS manifestations in the patient reported here, and KO mice do not display any NBCCS manifestations. Furthermore, none of the previously reported *PTCH2* individuals have been diagnosed with NBCCS according to one of the proposed sets of diagnostic criteria^3^. Finally, loss of function variants in *PTCH2* are frequent in population studies.

These observations question whether variants in *PTCH2* are associated with NBCCS; if, however, it is the case, the penetrance could be considered very low. Further studies of larger cohorts of individuals with *PTCH2* variants should clarify this.

## Supplementary information

Supplementary table

## Data Availability

The relevant data from this Data Report are hosted at the Human Genome Variation Database at 10.6084/m9.figshare.hgv.2534
